# Influence of Phthalates on Cytokine Production in Monocytes and Macrophages: A Systematic Review of Experimental Trials

**DOI:** 10.1371/journal.pone.0120083

**Published:** 2015-03-26

**Authors:** Juliana Frohnert Hansen, Klaus Bendtzen, Malene Boas, Hanne Frederiksen, Claus H. Nielsen, Åse Krogh Rasmussen, Ulla Feldt-Rasmussen

**Affiliations:** 1 Department of Medical Endocrinology, PE 2132, Rigshospitalet, University of Copenhagen, Copenhagen, Denmark; 2 Institute for Inflammation Research, section 7521, Rigshospitalet, University of Copenhagen, Copenhagen, Denmark; 3 Department of Growth and Reproduction, Rigshospitalet, University of Copenhagen, Copenhagen, Denmark; University of Leuven, Rega Institute, BELGIUM

## Abstract

**Background:**

Phthalates are a group of endocrine disrupting chemicals suspected to influence the immune system. The aim of this systematic review is to summarise the present knowledge on the influence of phthalates on monocyte and macrophage production and secretion of cytokines, an influence which could affect both pro- and anti-inflammatory abilities of these cells.

**Strategy and Results:**

A systematic search was performed in Medline, Embase and Toxline in June 2013, last updated 3rd of August 2014. Criteria used to select studies were described and published beforehand online on Prospero (http://www.crd.york.ac.uk/NIHR_PROSPERO, registration number CRD42013004236). *In vivo*, *ex vivo* and *in vitro* studies investigating the influence of phthalates on cytokine mRNA expression and cytokine secretion in animals and humans were included. A total of 11 reports, containing 12 studies, were found eligible for inclusion. In these, a total of four different phthalate diesters, six primary metabolites (phthalate monoesters) and seven different cytokines were investigated. Though all studies varied greatly in study design and species sources, four out of five studies that investigated di-2-ethylhexyl phthalate found an increased tumour necrosis factor-α secretion/production from monocytes or macrophages. A summary of cytokine measurements was not possible since few studies were comparable in study design and due to insufficient reporting of raw data for most of the included studies.

**Conclusion:**

Results from this review have suggested that at least one phthalate (di-2-ethylhexyl phthalate) has the ability to enhance tumour necrosis factor-α production/secretion from monocytes/macrophages *in vitro*, but also observed *ex vivo*. Influence of other phthalates on other cytokines has only been investigated in few studies. Thus, *in vitro* studies on primary human monocytes/macrophages as well as more *in vivo* studies are needed to confirm or dispute these findings.

## Introduction

In recent years, the effects of endocrine disrupting chemicals (EDCs) have been studied intensively. EDCs are defined as exogenous substances or a mixture thereof with ability to cause adverse health effects in the intact organism, its offspring or subpopulations, by altering functions of the endocrine system [1]. Phthalates are a group of endocrine disruptors, first produced in the 1920’s, and today hundreds of million tons are produced each year [2]. They are used mainly as plasticizers in materials such as polyvinyl chloride (PVC), but are added in the manufacturing processes of many other products as well. Phthalates are therefore found in many different end-products, such as building materials, paint, toys, perfume, personal care products and coating of drugs [3,4]. Phthalates are not chemically bound to the products and can leach into the environment. Consequently, they are also found in the indoor and external environments, for example in food, soil and water [3,5–7]. Constant exposure to phthalates through ingestion, inhalation and for humans through dermal absorption is therefore inevitable for both humans and wildlife. Some groups are more exposed to these chemicals than others. For instance, hospitalised patients are exposed through the use of infusion tubes and other medical devices, infants and toddlers through toys or ingestion of dust, and workers at plastic plants through their working environment [6,8,9]. The negative influence of phthalates, especially on the male reproductive system has given rise to restrictions in the use of these chemicals in children’s toys and child care products [8,10,11]. Other endocrine systems, such as the hypothalamo-pituitary-thyroid-axis, are also under investigation for potential influence by phthalates, reviewed in [12].

During the last decades of the 20th century, the use of chemicals such as phthalates gradually increased [2]. Concurrently, an increased prevalence of diagnosed allergy and eczema [13], as well as in diagnosed cases of some autoimmune diseases, was observed worldwide [14–16]. Although no evidence of a causal relationship exists, this gave rise to speculations that the immune system might be influenced by phthalates and or other EDCs. Epidemiological studies have investigated the possible correlation between asthma and respiratory symptoms and PVC or phthalate exposure in work places and homes, reviewed in [17,18]. Experimental studies, both in vivo and in vitro, have suggested that phthalates possess adjuvant effects on immunoglobulin production and influence the differentiation and cytokine secretion from T helper (Th) cells, enhance enzyme and histamine release, and increase phagocytic ability in cells from the innate immune system, reviewed in [18].

Monocytes and macrophages are innate immune cells. Monocytes circulate between the bone marrow and the blood stream. They can leave the circulation and invade tissues, for instance at a site of infection or other tissue damage, and there they differentiate into macrophages with a wide range of functions, including phagocytosis, antigen presentation to T lymphocytes, and secretion of hormone-like polypeptides and proteins (cytokines), including interferons and chemokines, by which macrophages recruit and modulate the functions of other immunoinflammatory cells [19]. Thus, macrophages are important inducers and modulators of inflammation, and they have important functions in host defence and tissue repair [20].

The aim of this review is to summarise what is known about the influence of phthalates (phthalate diesters and their metabolites) on cytokine production and secretion by monocytes and macrophages.

### Selected monocyte/macrophage-derived cytokines

Monocytes and macrophages produce a great number of cytokines and chemokines [21]. Of these, only tumour necrosis factor (TNF)-α, interleukin (IL)-1α and-1β, IL-6, IL-8/CXCL8, IL-10 and IL-12 will be described here, since only these were investigated in the studies included in this review.

TNF-α is an important proinflammatory cytokine secreted mainly by monocytes and macrophages but also by other cell types, including T cells, B cells and natural killer (NK) cells, as well as mast cells [21]. TNF-α plays an essential role in initiation of both local and systemic inflammation through induction of other cytokines (including IL-1β and IL-6 [22]), chemokines, inflammatory mediators and expression of endothelial cell adhesion molecules [23]. It also exhibits immunosuppressive properties, such as inhibition of T-cell signalling and reduction of the antigen presentation by dendritic cells [22]. TNF-α is important in defending the host against pathogens and in maintenance of the micro-architecture of secondary lymphoid tissue (reviewed in [23]). It is a main actor in the pathogenesis of septic shock, and plays a pathogenic role in several autoimmune and inflammatory diseases [22,24]. The vast majority of investigations make use of bacterial lipopolysaccharide (LPS) as inducer of TNF-α and other pro-inflammatory cytokines, as LPS is a powerful activator of the innate immune system (primarily monocytes/macrophages) [21].

IL-1α and IL-1β are proinflammatory cytokines with similar activities, as they function through the same set of IL-1 receptors on a large number of target cells. IL-1α is primarily produced by monocytes/macrophages, endothelial cells and keratinocytes [25]. IL-1β is produced as pro-IL-1β mainly by monocytes/macrophages, dendritic cells, B cells and NK cells [26]. The production/secretion of both cytokines is induced by LPS and augmented by the cytokines themselves through auto- and paracrine mechanisms [21]. Upon cell death, intracellular IL-1α is released and may cause inflammation, since its precursor is biologically active. IL-1α is also important for interferon (IFN)-γ-mediated activities [26]. Pro-IL-1β needs to be cleaved to become biologically active. This is achieved by the engagement of the inflammasome, which in turn is activated by danger molecules or substances such as ATP or cholesterol [26,27]. IL-1β induces local inflammation and often also systemic signs of inflammation (fever and augmented acute-phase protein production). Directly or indirectly, IL-1β also induce production of adhesion molecules and various enzymes, such as cyclooxygenase type 2, and, as mentioned above, increased production of various other cytokines including chemokines [26]. IL-1β also influences adaptive immune responses by affecting both T cells and B cells, e.g. it plays a role in Th17-cell development, and the cytokine seems to have pathogenic importance in certain autoinflammatory diseases [26].

IL-12 is a proinflammatory cytokine secreted mainly by monocytes/macrophages, dendritic cells and B cells [28], and LPS and other components of microorganisms are important inducers of the cytokine [21]. IL-12 directs differentiation of T cells towards a Th1-profile characterised by production of IFN-γ [28]. Also the IFN-γ secretion by NK cells and their cytotoxic ability is stimulated by IL-12 [28]. In vivo, IL-12 plays an important role in fighting intracellular pathogens, among other functions [28].

IL-8 or CXCL8 is classified as an inflammatory chemokine, and is in human secreted by many cell types, including monocytes/macrophages, fibroblasts, endothelial and epithelial cells [21]. The secretion of CXCL8 by monocytes/macrophages is induced by LPS, but also by proinflammatory cytokines such as IL-1β and TNF-α [21]. Chemokines are smaller than cytokines, containing about 70 to 130 amino acids, and they act locally through paracrine or autocrine cell signalling [29]. CXCL8 directs the migration of neutrophils and influences exocytotic functions of leukocytes, for example secretion of proteases and cytotoxic proteins [29].

IL-6 has both pro- and antiinflammatory/regenerative abilities. IL-6 is produced by almost all cell types, including monocytes/macrophages, T cells, B cells, endothelial cells and keratinocytes [21], but also by classical endocrine cells such as thyroid, pituitary, parathyroid and adrenocortical cells [30–33]. In monocytes, IL-6 secretion is induced by TNF-α and IL-1α and-β, as well as by endotoxins and viral infections [21]. By influencing the secretion of chemokines, IL-6 is involved in the switch from neutrophil to monocyte recruitment to a site of acute inflammation [34]. It is also active in T-cell recruitment and differentiation of both T cells and B cells [34]. IL-6 is an important inducer of the acute-phase reaction including production of acute-phase proteins in the liver [21].

IL-10 is an anti-inflammatory cytokine secreted by monocytes/macrophages, as well as by T cells and B cells [35]. Its secretion by monocytes/macrophages is stimulated by LPS [21] and by catecholamines [35]. IL-10’s anti-inflammatory actions on monocytes and macrophages are mediated through inhibition of production of various proinflammatory cytokines, inhibition of antigen-presentation and enhancement of phagocytosis [35]. Also, secretion of pro-inflammatory cytokines by Th cells and secretion of inflammatory mediators by eosinophil and neutrophil granulocytes are inhibited by IL-10 [35]. Proliferation and expression of the major histocompatibility complex (MHC) class II molecules by B cells are enhanced by IL-10 [35].

### Selected phthalates

Many different phthalates are used by the industry. Only a minor selection of these has been investigated in monocytes and macrophages, and thus only these phthalates are further described in this review.

Phthalates are lipophilic, but are metabolised to become more hydrophilic [36]. Through the action of non-specific enzymes, they are rapidly hydrolysed from phthalate diesters to the respective primary metabolite (monoesters). Short-branched phthalates are mainly excreted in urine as monoesters, while long-branched phthalates undergo further oxidation before they are excreted as secondary partly glucuronidated oxidative metabolites [36–38]. Secondary metabolites have longer half-lives than primary metabolites [37–39]. Children seem to have a more efficient oxidative metabolism, and new studies show that preterm and healthy infants are more exposed and have significantly different phthalate metabolism compared to older children and adults [9]. Bio-monitoring phthalate exposure is normally done by assessing metabolite content in urine. However lower amounts (10–100 fold) of especially monoesters and secondary carboxylated metabolites are present in serum [40–42].

Di-butyl phthalate covers two isoforms; di-n-butyl phthalate (DnBP) and di-iso-butyl phthalate (DiBP) and are low-molecular weight phthalates, often used together with high-molecular ones. DnBP is mostly used as a plasticizer, i.e. plastic softener, but also as a solvent in dyes and nitrocellulose paints, ink, pesticides, adhesive agent in glue, fragrance solvent, softener in cosmetic products and nail-polish and as coating of enteric medication [43,44]. Through regular intake of pills, a 100 times higher DnBP exposure was observed compared to DnBP levels in the general population [45]. DiBP is often used in mixtures with or as a substitute to DnBP [41]. Both DBP isomers are mainly excreted as glucuronidated monoesters (mono-n-butyl phthalate (MnBP) and mono-iso-butyl phthalate (MiBP)), while minor amounts are excreted as secondary metabolites [46].

Di-2-ethylhexyl phthalate (DEHP) is a high-molecular weight phthalate and is one of the most commonly used phthalates. DEHP is mostly used as a plasticizer in PVC manufacturing. PVC is, in turn, used in building materials (roof and wall coverings, floor tiles, isolation and sealing pastes, cable isolation), clothes and shoes (rain coats, rubber boots, out-door clothes, garment made of imitated leather), packing films, toys and medical products (including disposable gloves). A small proportion of DEHP is used in non-PVC related products as additives to rubber, latex, ink, pigments and lubricants [47,48]. Compared to the low-molecular weight phthalates, DEHP is mainly excreted in urine as secondary metabolites. DEHP is rapidly hydroxylated to the primary metabolite mono-(2-ethylhexyl) phthalate (MEHP) and the major part is further oxidised to secondary metabolites with functional hydroxyl-, oxo- and carboxy groups—more or less glucuroniadated [49,50].

Di-iso-nonyl phthalate (DiNP) and di-iso-decyl phthalate (DiDP) are long branched mixtures of different dinonyl and didecyl isomers. In humans the urinary excretion pattern of DiNP and DiDP metabolites are similar to that of DEHP [39,51,52]. Both DiNP and DiDP are used in different mixtures as replacements for DEHP [53] and the consumption has increased over recent years [41,54–56].

Butylbenzyl phthalate (BBzP) is a high-molecular weight phthalate with an asymmetrical structure. About half of the produced BBzP is used as plasticizer in PVC products, the rest is used in other products such as sealants, adhesives, paints, inks and lacquers [57,58]. BBzP is metabolised to both mono-benzyl phthalate (MBzP) and MnBP [59].

## Methods

The PRISMA statement for reporting systematic reviews [60] and Cochrane Handbook for systematic reviews of interventions (http://handbook.cochrane.org) were used as guidance in the process of this systematic review (S1 PRISMA Checklist).

### Study and report criteria

A protocol is registered online at Prospero (http://www.crd.york.ac.uk/NIHR_PROSPERO, registration number CRD42013004236), which describes study and report criteria used to select studies. Shortly, only published/e-published English language reports were included. In vivo, ex vivo or in vitro studies were included, with no restriction of study design. Study subjects had to be human or animal monocyte or macrophage cell lines or primary cell cultures, activated with LPS if they consisted of mixtures of cells. These had to be exposed to phthalates (di-ester, mono-ester or secondary metabolites), either orally or by inhalation, intra dermal, intra peritoneal or intra venous routes. Controls had to be maintained or cultured under the same condition, but exposed to vehicle without phthalates. The primary outcome was change in cytokine protein secretion and/or mRNA expression, and the secondary outcome was mortality, morbidity or cell death.

### Database search and study selection

A systematic search was performed electronically in Medline (1946 to present, provider: Ovid), Embase (1974 to present, provider: Ovid) and Toxline (provider: US National Library of Medicine). The databases were searched on the 6^th^ of February 2013 (Medline), 8^th^ of February 2013 (Embase) and 12^th^ to 13^th^ of February 2013 (Toxline). The same search was redone in all databases on the 19^th^ (Medline) and the 20^th^ of June 2013 (Embase and Toxline), since the process of screening search results was delayed. An updated search was performed on 3^rd^ of August 2014 in Medline and Embase. Search terms were Phthalat*; Phthalic acids; Plasticizer; Leukocyte; Leucocyte; Monocyte; Monocytes; Mononuclear cells; Macrophages; Interleukin; Cytokine; Interferon and Inflammasome (S1 Text).

Title and abstract were screened for potentially relevant reports, which were read in full text. If they matched the inclusion criteria, data was extracted. This process was done independently by two review authors, Juliana Frohnert Hansen (JFH) and Ulla Feldt-Rasmussen (UFR). Results from the data extraction were compared and any disagreement was resolved by discussion with a third review author, Klaus Bendtzen (KB).

### Data extraction and analysis

Data was extracted using a data-extraction sheet developed by JFH, which was pilot-tested in three different studies and adjusted before use. The following information was sought in the process of extracting data: study design, participant characteristics, details of exposure in control and intervention groups, methods used to asses both primary and secondary outcomes, summary data or raw data to calculate summary data from each intervention group, and information to assess risk of bias. If necessary information for the study was not found, review authors had to contact the study authors. To identify possible multiple reports, interventions and outcome were compared, if author(s) and study participants were the same.

Risk of bias assessment was planned to be used as a qualitative judgment of the individual studies included in this review, with the Cochrane Collaboration’s tool for assessing risk of bias when possible.

Summary measurement for the primary outcome was beforehand stated to be presented as mean, standard deviation (SD) and range, and not further specified for the secondary outcome.

## Results

### Study selection

**Fig 1:**
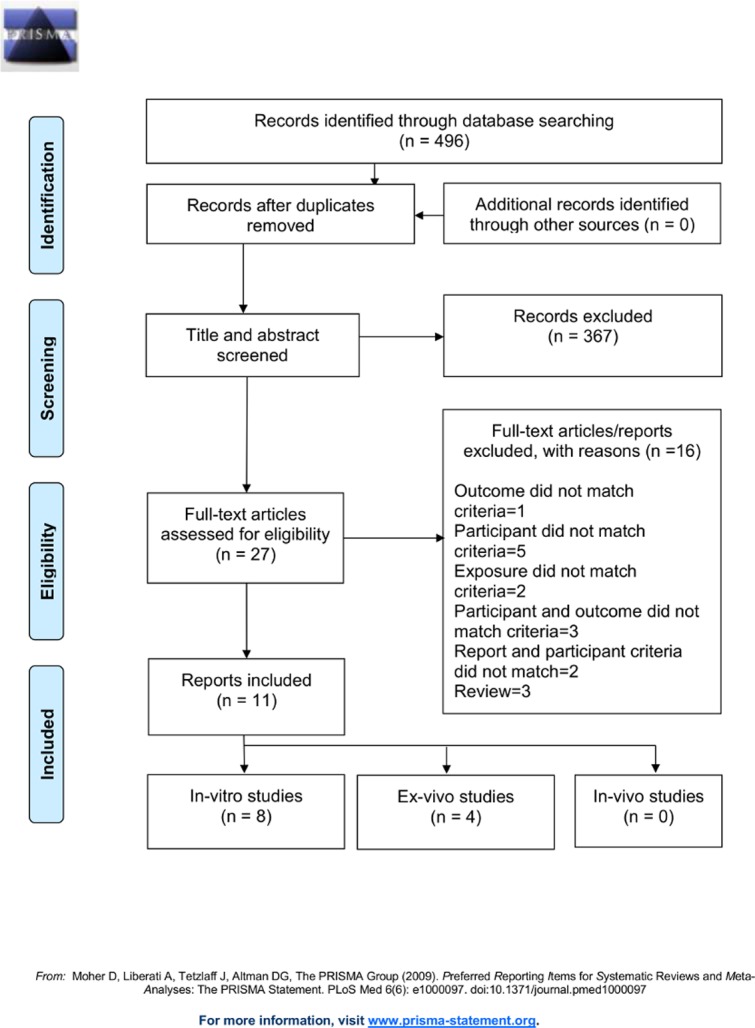
PRISMA flow chart.

A total of 496 records were identified searching the three databases, and 394 remained after removal of duplicates. A total of 367 reports were excluded, and the remaining 27 were read in full text and assessed for eligibility. Sixteen of the 27 reports did not match participant, exposure or outcome criteria and were excluded. A total of 11 reports remained and were included in the review (Fig. 1). No duplicate publications were found amongst these, and no additional articles were found from reference lists of either potential or relevant reports that were read in full text. In the updated search from the 3^rd^ of August 2014, seven new potential reports were assessed for eligibility, but did not match participant criteria.

### Study characteristics

Details of the individual study characteristics are found in S1 Table. An overview is given in Table 1.

**Table 1:** Study characteristics.

Source	Type of study	Study design	Participants	Cell/tissue studied	Phthalate exposure	Phthalate Concentration	Frequency and duration of exposure
Zheng 2010 [63]	Ex vivo	RCT	Sprague Dawly rats, male	Testicular macrophages and testes homogenates (no cultures but paraffin embedded and frozen tissue)†	DnBP	50 and 250 mg/kg/day	Once daily by gavage, for totally 90 days, outcome asssessed at 30 and 90 days
Tonk 2012 [65]	Ex vivo	NRCT	Wistar rats (male)	Adherent splenocytes (directly after in-vivo exposure) *	DEHP	1, 3, 10, 30 100, 300, 1000 (only adult) mg/kg/day	Daily for 40 days by gavage
Piepenbrink 2005 [62]	Ex vivo	RCT	CD-strain Sprague-Dawley rats (female)	Adherent spleocytes (taken out 5 weeks and 13 weeks after exposure/birth) *	DEHP	37.5, 75, 150, 300 mg/kg	Daily for 16 days by gavage
Hong 2004 [61]	Ex vivo	NRCT	Balb/c mice, (female)	Abdominal macrophages, cells obtained 24h after phthalate exposure *	BBzP	1 mg/0,1ml/mouse	Once, subcutaneous
Hong 2004 [61]	In vitro	-	Mouse	Raw 264 cell line	BBzP	10 μg/ml	Once, incubation 4h
Nishioka 2012 [71]	In vitro	-	Human	THP-1 cell line differentiated by PMA-stimulation	DEHP	2*10^-4^M	Once. incubation for 8h (3 h followed by 5h +/-zymosanA)
Bennasroune 2012 [69]	In vitro	-	Human	THP-1 cell line differentiated by PMA-stimulation	DiNP	0.2, 2, 5 and 10 μM	Once, incubation for 24 h
Glue 2002 [70]	In vitro	-	Human	THP-1 cell line	MnBP, MBzP, MEHP, MOP, MiNP, MiDP	0.2, 2, 20, 200 μg/ml (toxicity). 2 and 20 μg/ml (cytokine mRNA)	Once, incubation 24h
Kocbach 2012 [68]	In vitro	-	Mouse	Raw 264.7 cell line	MEHP	5*10^-5^, 10^-4^, 3*10^-4^, 5*10^-4^and 10^-3^ M	Once, incubation for 3h
Li 2013 [64]	In vitro	-	Mouse	Murine peritoneal exudate macrophages†	DnBP	10^-6^, 5*10^-6^, 10^-5^, 5*10^-5^ and 10^-4^ M (cytotoksitet), 10^-6^ and 10^-5^ M (cytokine+mRNA)	Once, incubation for 24h
Yamashita 2005 [67]	In vitro	-	Balb/c mice, female	Thioglycolate-induced peritoneal exudate macrophages†	DBP	10^-6^, 10^-7^, 10^-8^ M (IL-1 α), 10^-7^ M (IL-6, IL-12, TNF-α)	Once, incubation for 24h
Yamashita 2002 [66]	In vitro	-	Balb/c mice, female	Thioglycolate-induced peritoneal exudate macrophages†	DEBP	10^-7^ M	Once, incubation for 2 days.

Footnote:

*: macrophage/monocyte content not confirmed, but cells stimulated with LPS.

†: macrophage content confirmed by flow cytometry.

BBzP: Butylbenzyl phthalate, DEHP: Di-2-ethylhexyl phthalate, DiNP: Di-iso-nonyl phthalate, DnBP: Di-n-butyl phthalate, IL: interleukin, MBzP: Mono-benzyl phthalate, MEHP: Mono-(2-ethylhexyl) phthalate, MiDP: Mono-iso-decyl phthalate, MiNP: Mono-iso-nonyl phthalate, MnBP: Mono-n-butyl phthalate, MOP: Mono-n-octyl phthalate, NRCT: Non randomised controlled trial, PMA: Phorbol 12- myristate 13-acetate, RAW 264 cell line: mouse leukemic monocyte-macrophage cell line, RCT: Randomised controlled trial, THP-1 cell line: acute monocytic cell line, TNF: tumour necrosis factor.

One of the 11 included reports consisted of both an in vitro and an ex vivo study [61], resulting in a total of 12 studies. Eight were in vitro studies and four ex vivo studies. Of the ex vivo studies, only two were randomised trials [62,63]. The other two and all in vitro studies had a control group.

Nine of the included studies used animal tissue as their study participant material, and seven used primary cells from mice or rats [61–67]. Four of these studies confirmed the macrophage content by flow cytometry [63,64,66,67], and the remaining three studies used LPS as a monocyte/macrophage specific activator [61,62,65]. Two studies used the Raw 264 murine cell line (mouse leukemic monocyte-macrophage cell line) [61,68]. A total of three studies used human tissue, in all cases the THP-1 cell line (acute monocytic cell line) [69–71], which was differentiated to macrophages by phorbol 12-myristate 13-acetate (PMA)-stimulation in two of the studies [69,71].

**Fig 2:**
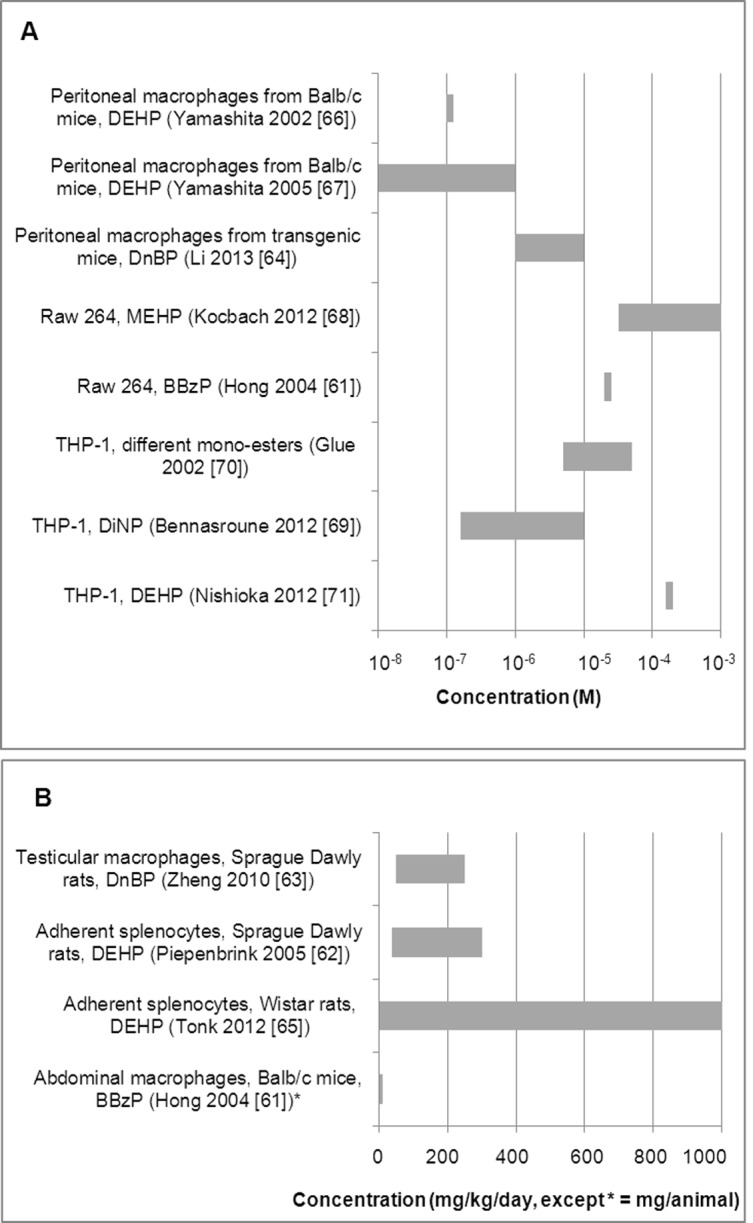
Phthalate concentration range used in the included in vitro (A) and ex vivo (B) studies.

**Fig 3:**
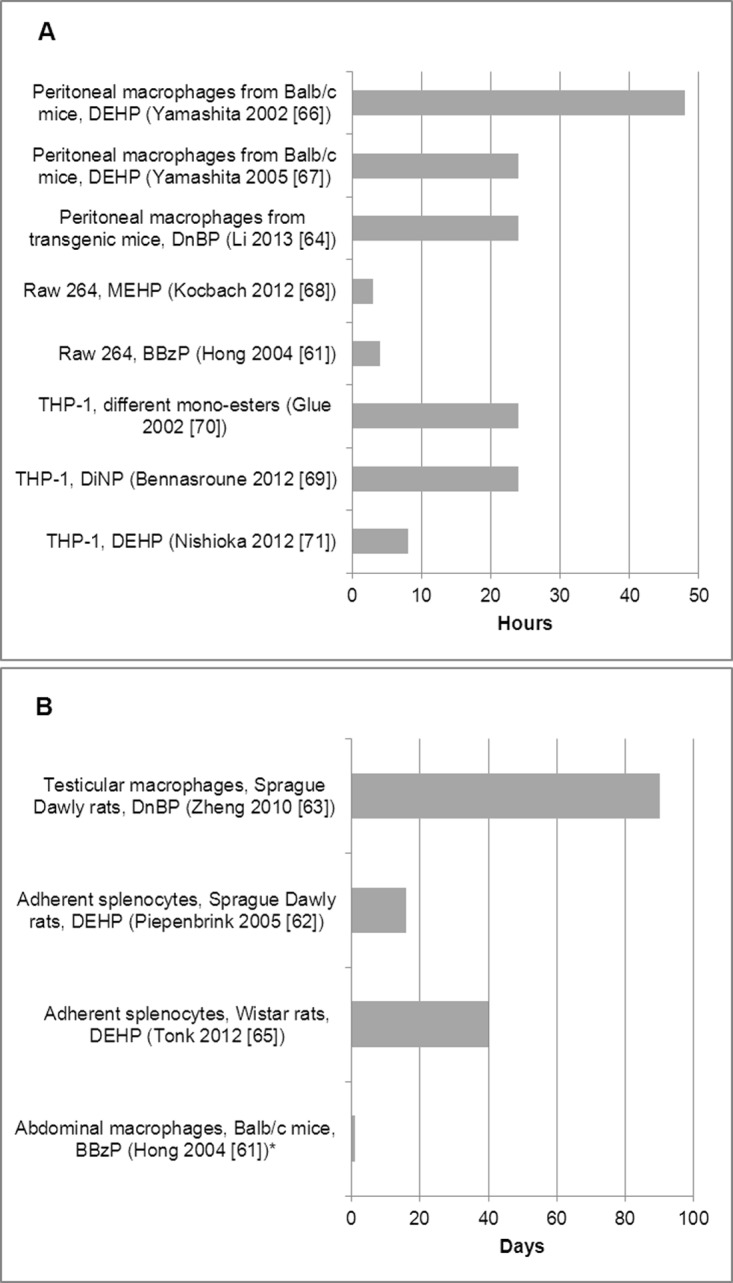
Study duration used in the included in vitro (A) and ex vivo (B) studies.

Ten of the included studies exposed cells or participants to phthalate diesters (DiNP, DEHP, DnBP or BBzP) [61–67,69,71], and two used primary metabolites (MEHP, MnBP, MBzP, mono-n-octyl phthalate (MOP), mono-iso-nonyl phthalate (MiNP) or mono-iso-decyl phthalate (MiDP)) [68,70]. DEHP and its primary metabolite MEHP were studied more often than the other phthalates. The phthalate concentration range used in all the included studies was broad, ranging from 10^-8^ to 10^-3^ M for in vitro studies, and from 1 to 1000 mg/kg/day for ex vivo studies. Ranges were not always overlapping in studies that used similar cell cultures (Fig. 2 A and B). Although the study duration varied, especially among the ex vivo studies, it was comparable in most of the in vitro studies that used similar cell cultures (Fig. 3 A and B).

All the included studies used a control group. Phthalates are usually dissolved in either ethanol or dimethyl sulfoxide (DMSO). In one study, it was both unclear what the phthalate was dissolved in and what the control group received [69], and in four other studies it was unclear what the control had received [61,64,66]. Another study exposed the control group to phosphate buffer saline (PBS) but not to ethanol [67].

### Risk of bias within studies

As mentioned earlier, only two of the included studies were randomised trials. Instead of the Cochrane Collaboration’s tool for assessing risk, comments from review authors on randomisation, possible attrition and reporting bias, as well as the studies’ funding sources were used (S1 Table). In general, information on excluded participants/cell cultures and outcome, was poor or not given at all in the published reports. None of the included studies provided information on possible confounders, and were therefore not controlled for. Details of how the randomisation was performed, was not given in either of the two randomised trials. Funding sources were not given in three studies [65,66,69].

### Results of individual studies: Cytokine secretion/production

The primary outcome was change in cytokine secretion and/or cytokine mRNA expression in the intervention group compared to control group (Table 2 and S1 Table).

**Table 2:** Primary and secondary outcomes from individual studies.

Source	LPS used	Primary outcome: cytokine secretion/production	Secondary outcome: toxicity of phthalates
Testicular macrophages, Sprague Dawly rats, DnBP (Zheng 2010 [63])	No	↑ IL-1 β	ND
Adherent splenocytes, Wistar rats, DEHP (Tonk 2012 [65])	Yes, 15 μg/ml	↑ TNF-α in adult group. ↔ TNF-α in juvenil group.	ND
Adherent splenocytes, Sprague Dawly rats, DEHP (Piebenbrink 2005 [62])	Yes, 10 ng/ml (incubation ex-vivo for 24 hours)	↔ TNF-α (5 weeks after exposure ended)	Still birth and early death of pups were low, results given in mean +/- SEM. Death in parental group not specified. Cell toxcicity ND.
Abdominal macrophages, Balb/c mice, BBzP (Hong 2004 [61])	Yes, 1 og 10 ng/ml (incubation ex-vivo for 4 hours)	↔ TNF-α	Unclear if assessed
Raw 264 cell line, BBzP (Hong 2004 [61])	Yes, 10 ng/ml (incubation together with phthalates)	↓ TNF-α	No data (crystal violet staining), conclusion is no cytoxcicity
THP-1 cell line, DEHP (Nishioka 2012 [71])	No	↑ TNF-α, IL-1 β and IL-8, ↔ IL-6 (mRNA expression: ↑ TNF-α, IL-1 β, IL-8 and IL-6)	ND
THP-1 cell line, DiNP (Bennasroune 2012 [69])	Yes, 5ng/ml (cell viability) or 10 ng/ml (cytokine secretion) (administred 24h after phthalate exposure and incubation for another 24h)	↑ TNF-α, ↔ IL-1 β and IL-8	Cell viability, <10% decrease
THP-1 cell line, different monoesters (Glue 2002 [70])	Yes, 1 μg/ml (only cytokine assessment)(incubation together with phthalates)	↔ IL-1 β, IL-6, IL-12 α p35 (mRNA epxression)	Live cells per ml (tryphan blue), leathal phthalate monoester concentration when higher than 20 μg/ml.
Raw 264 cell line, MEHP (Kocbach 2012 [68])	No	↑ TNF-α	No data (PI/Hoechst staining, LDH), conclusion: no significant increase in toxcicity.
Peritoneal macrophages from transgenic mice, DnBP (Li 2013 [64])	Yes, 100 ng/ml (only cytokine assessement) (incubation 6h after phthalates exposures)	↓ TNF-α, IL-1 β and IL-6, ↔ IL-10 and IL-12 (both cytokine secretion and mRNA exression)	Data only in graph, mean +/- SD (tryphan blue), data as a representative flowcytometry sample with percentage of positiv cells (PIannexin V-staining). Conclusion: 5*10^-5^ M and 10^-4^ M significant cytotoxcity
Peritoneal macrophages from Balb/c mice, DEHP (Yamashita 2005 [67])	No	↑ TNF-±, IL-1 ±, IL-6 and IL-12	ND
Peritoneal macrophages from Balb/c mice, DEHP (Yamashita 2002 [66)	Yes, 5 μg/ml (incubation 24 h after phthalate exposure)	Without LPS: ↑ TNF-± and IL-1 ±, With LPS: ↑ IL-1 ±, ↔ TNF-±	ND

Footnote: BBzP: Butylbenzyl phthalate, DEHP: Di-2-ethylhexyl phthalate, DiNP: Di-iso-nonyl phthalate, DnBP: Di-n-butyl phthalate, IL: Interleukin, LDH: lactate dehydrogenase, MEHP: Mono-(2-ethylhexyl) phthalate, ND: not done, PI: Propidium Iodide, RAW 264 cell line: mouse leukemic monocyte-macrophage cell line, SD: standard deviation, SEM: standard error of the mean, THP-1 cell line: acute monocytic cell line, TNF: Tumour necrosis factor.

TNF-α was the most commonly assessed cytokine, followed by IL-1β, IL-6, IL-12, IL-1α, CXCL8 and IL-10. Cytokine secretion was assessed in eleven studies and two of these also assessed cytokine mRNA expression [64,71]. A third study by Zheng et al. [63], also assessed mRNA secretion, though this was done on testes homogenates and not specific macrophages, so these results were not included in the review [63]. One study only assessed mRNA expression [70].

All the included studies differed in design. In the process of data extraction, an attempt was made to collect simple summary or raw data to calculate mean and SD for the primary outcome. Most of the papers only reported them in graphs with insufficient raw data provided to calculate summary data by the review authors. Since few studies would be comparable in study design, no further attempt was made to contact authors to collect data.

The only two studies comparable in study design (comparable in participants/cell cultures, phthalate exposure and study duration) came from the same study group and had similar results: DEHP increased TNF-α and IL-1α secretion from murine peritoneal macrophages (without LPS-stimulation) [66,67]. One of these studies also investigated IL-6 and IL-12, the secretion of which was also increased by DEHP [67]. DEHP had similar effects on the human THP-1 cell line in a concentration at least 200 times higher (2*10^-4^ M) and with shorter incubation time: an increase in the secretion of TNF-α, IL-1β and CXCL8, but not of IL-6 (without LPS-stimulation). This was confirmed by quantitative real-time polymerase chain reaction (qRT-PCR), where mRNA expression of all the four cytokines was increased by DEHP [71]. The influence of DEHP in two ex vivo studies was investigated in murine adherent splenocytes stimulated with LPS. Again an increase in TNF-α was found [65], though this could not be detected after a five week restitution period [62].

DnBP was investigated in two studies with contradicting results. DnBP increased IL-1β in murine testicular macrophages (ex vivo study without LPS-stimulation) [63], but suppressed secretion of IL-1β, TNF-α and IL-6 in LPS-stimulated murine peritoneal macrophages (in vitro study with transgenic mice), which was also confirmed by mRNA assessment of the same cytokines [64].

Another phthalate, BBzP, also suppressed the LPS-stimulated TNF-α secretion from the murine monocyte/macrophage leukaemic cell line (RAW 264), though this could not be confirmed in an ex vivo study by the same study group [61].

DiNP increased TNF-α secretion from LPS-stimulated THP-1 cells, but had no influence on IL-6 or CXCL8 secretion [69]. No other phthalates were investigated in this study.

Two studies investigated the primary metabolites, though the outcome was not comparable. Glue et al. found no influence by several different primary metabolites on the mRNA expression of IL-1β, IL-6 and IL-12 α p35 in human THP-1 cells [70]. Kocbach et al., however, found an increase in TNF-α secretion by MEHP in the murine RAW 264 cell line [68].

### Results of individual studies: Toxicity of phthalates

The secondary outcome was mortality or morbidity of study subjects or cell death compared to control (Table 2 and S1 Table).

One of the four ex vivo studies assessed mortality of study participants, though not all intervention groups were mentioned [62]. Still birth and early deaths were low for offspring of DEHP exposed rats in that study. Though another ex vivo study, investigating DEHP in juvenile rats, did not assess mortality, the highest exposure group was excluded, due to high pup deaths [65]. The dose of DEHP in the latter study was over 3 times higher than the highest dose of the former study. A third ex vivo study may also have assessed mortality, but the published report is unclear at this point [61].

Five of the eight in vitro studies assessed cell toxicity or cell death [61,64,68–70]. Different phthalates and primary metabolites were investigated in either primary murine cell cultures, and in murine or human cell lines, and results are therefore difficult to compare. Two studies used the human THP-1 cell line, and found different primary metabolites (including MiNP) toxic at concentrations higher than 20 μg/ml (6.9*10^-5^ M for MiNP) [70], and DiNP non-toxic at up to 10^-5^ M [69]. Phthalate exposure time was 24 hours in both studies. Two other studies used the murine RAW 264 cell line, and found neither BBzP [61] nor MEHP [68] toxic at concentrations 3*10^-5^ M and 5*10^-5^ to 10^-3^ M, respectively. Phthalate exposure time was four and three hours, respectively. The fifth study used murine peritoneal macrophages and found DnBP toxic at 5*10^-5^ M and 10^-4^ M [64]. Phthalate exposure time was 24 hours.

The studies that found some of the phthalate concentrations toxic did not assess cytokine secretion or mRNA expression at the same concentrations.

## Discussion

In general, few published studies have investigated the influence of phthalates on macrophage cytokine secretion. Murine cells or cell lines or human cell lines were used making it difficult to interpret results and their possible significance for healthy human individuals. One report from the database research used human peripheral blood mononuclear cells, but was not included since no stimulus was used for macrophage activation [72].

DEHP, the most studied phthalate in relation to monocyte/macrophages cytokine secretion, was used in five out of 12 studies [62,65–67,71]. Although the study design varied greatly among these, the outcome was comparable in one sense: DEHP enhanced TNF-α secretion by monocytes/macrophages in four out of five studies. The one study where no influence was found, participants (female rats) were exposed in utero (mothers were exposed by gavage) and TNF-α was assessed after a five week restitution period [62]. The other ex vivo study of DEHP exposure by gavage in both juvenile and adult male rats, used similar exposure doses but the TNF-α secretion was only influenced in macrophages from adult rats [65]. This might suggest that monocytes/macrophages from rat foetuses and juvenile rats were less sensitive to the influence by DEHP, though a non-persistent influence by DEHP in the former study also seemed plausible as well as an influence in male rats only. DEHP concentration in the in vitro study on the human THP-1 cell line was higher and duration longer compared to the two in vitro studies on primary murine peritoneal macrophages. Still the same influence on TNF-α was found, indicating that cell lines are less sensitive than primary cell cultures, but also suggested that the same pathway was involved. Difference in number of expressed receptors or in ligand affinity to the receptor might cause cell lines and primary cell cultures to react differently, an observation made previously with thyroid cell cultures [73]. Pathways were investigated in both ex vivo studies [62,65] (NO/nitrite production) and one of the in vitro studies [71] (activation of nuclear factor-kappa B (NF-κB)), and suggests NF- κB to be involved as well as NO, though the latter was not significant in any of the two ex vivo studies.

None of the three in vitro DEHP-studies investigated the toxicity of DEHP on cell cultures, an issue which should be addressed in future studies. Other phthalates than DEHP were toxic at concentrations 5*10^-5^ M or higher, at least when cells were exposed for 24 hours [64,69,70]. DEHP’s ability to enhance TNF-α secretion was found at a concentration of 2*10^-4^ M but also at 1000 times lower (10^-7^ M) concentrations [67].

For other phthalates, often just a single study was found, or if two studies were found, production of different cytokines was measured. Despite this, it is noteworthy that DiNP and MEHP may also enhance TNF-α secretion [68,69]. This is worrying, since DiNP is used as a replacement for DEHP [53]. BBzP and DnBP might inhibit TNF-α secretion, an inhibition not caused by cytotoxicity [61,64]. These results could indicate that other phthalates than DEHP can influence TNF-α secretion, but also that different phthalates could have opposite influences on cytokine secretion. To confirm these findings, future studies preferable using both mice and human cell cultures should be performed.

Despite the fact that the influence of DEHP on TNF-α secretion was the same both ex vivo and in vitro, similar findings were not seen for the influence of BBzP or DnBP on TNF-α and IL-1β production, respectively [61,63,64]. IL-1β production was stimulated ex-vivo but supressed in vitro by DnBP, probably reflecting additional in vivo mechanisms/pathways. Hong et al. studied BBzP’s influence on TNF-α both in female mice and a murine cell line, but found an influence only in the cell line [61]. However, a single subcutaneous exposure with a lower dose was given, which differed from the other ex vivo studies [62,63,65] where exposure was oral by gavage for 16 days or longer. This might explain why the in vitro results were not confirmed ex vivo.

Several other cytokines than TNF-α were assessed in the reviewed studies, but study designs varied too much and studies were too few to make sustainable conclusions from these results.

The toxic phthalate concentrations in the included studies were rather high, and probably higher than most individuals are exposed to in everyday life [9,74–77]. Also, in the included studies reviewed here, the phthalates were always investigated separately, which does not reflect the simultaneous exposure to multiple EDCs occurring in vivo.

Recently, researchers have become more aware of the possible synergistic effects of EDCs, also called the “cocktail effect”. Studies have shown that single substances without any harmful effect at low concentrations become harmful at the same low concentrations when mixed with other EDCs [78].

Two studies did not reveal the number of participants or cell cultures that the calculations were based on [62,68]. In three other studies, it was unclear if the results came from triplicate, duplicate, or single determinations [63,69,70]. Therefore, a statistical type 2 error may have occurred in these studies.

### Limitations

As mentioned earlier, only two randomised studies existed in the literatures and were included in this review [62,63]. Unfortunately, type of randomisation, sequence and allocation concealment and information on blinding were not given in these reports.

Concerning attrition bias, the reason for exclusion and the number of excluded cell cultures and their outcome results were usually not reported in the in vitro studies. Circumstances unrelated to the phthalate exposure could lead to exclusion in both control and intervention groups, and should, in general, not cause bias. Exclusion, even if none was made, should, however, be mentioned in the report, which was often not done, neither in the in vitro nor the ex vivo studies.

The review authors found signs of reporting bias in some studies [64,67,68], where e.g. results were only mentioned in the discussion of the report [67], or a non-toxic phthalate concentration was excluded in subsequent experiments without explaining why this was done [64]. A suggested dose-response effect could therefore be false.

Due to the above mentioned limitations, a systematic evaluation of the quality among the included studies was not possible.

## Conclusion

Evidence from published studies has suggested that at least DEHP enhances TNF-α secretion from monocytes/macrophages in vitro, which was also found in vivo. However, the low number of reports and great differences in study design generally precludes conclusions concerning the influence of other phthalates than DEHP or production of other cytokines than TNF-α. In general, more studies are needed to confirm or dispute these findings especially more in vivo studies and studies on primary human monocytes/macrophages to evaluate the relevance for humans. Also, the cocktail effect of low dose phthalate exposure should be part of the design of future studies.

## Supporting Information

S1 PRISMA Checklist (DOC)

S1 Table Study characteristics and outcome—detailed information. Footnote: *: macrophage/monocyte content not confirmed, but cells stimulated with LPS. †: macrophage content confirmed by flow cytometry. BBzP: Butylbenzyl phthalate, DEHP: Di-2-ethylhexyl phthalate, DiNP: Di-iso-nonyl phthalate, DMSO: dimethyl sulfoxide, DnBP: Di-n-butyl phthalate, IL: interleukin, IOD: integrated optical density, LDH: lactate dehydrogenase, LPS: lipopolysaccharide, MBzP: Mono-benzyl phthalate, MEHP: Mono-(2-ethylhexyl) phthalate, MiDP: Mono-iso-decyl phthalate, MiNP: Mono-iso-nonyl phthalate, MnBP: Mono-n-butyl phthalate, MOP: Mono-n-octyl phthalate, ND: not done, NRCT: Non randomised controlled trial, PBS: phosphate buffer saline, PMA: Phorbol 12-myristate 13-acetate, PND: postnatal day, qRT-PCR: quantitative real time polymerase chain reaction, RAW 264 cell line: mouse leukemic monocyte-macrophage cell line, RCT: Randomised controlled trial, rt-PCR: real time polymerase chain reaction, SD: standard deviation, SEM: standard error of the mean, THP-1 cell line: acute monocytic cell line, TNF: tumour necrosis factor.
(XLSX)

S1 Text Full search strategy. (DOCX)
