# Supplementary material for: Influence of Phthalates on Cytokine Production in Monocytes and Macrophages: A Systematic Review of Experimental Trials
Source: PLoS One. 2015 Mar 26;10(3):e0120083. doi: 10.1371/journal.pone.0120083 (PMC4374770; doi:10.1371/journal.pone.0120083)
Supplement: S1 Text — (DOCX) [file pone.0120083.s003.docx]

**S1 Text. Full search strategy.**

Searches were developed by Klaus Bendtzen (KB) and Juliana Frohnert Hansen (JFH).

JFH conducted all database searches and removed duplicates.

Medline 1946- (provider: Ovid).

Start and end date of search: 6^th^ of February 2013 (331 reports found).

Renewed search: 19^th^ of June 2013 (381 reports found).

Updated search: 3^rd^ of August 2014 (372 reports found).

1. Phthalat*.mp.

2. exp Phthalic acids/

3. exp Plasticizers/

4. plasticizer.mp.

5. 1 or 2 or 3 or 4

6. exp Leukocytes/

7. Leukocyte.mp.

8. Leucocyte.mp.

9. Monocyte.mp.

10. Monocytes.mp.

11. exp Monocytes/

12. mononuclear cells.mp.

13. exp. Macrophages/

14. macrophages.mp.

15. 6 or 7 or 8 or 9 or 10 or 11 or 12 or 13 or 14

16. exp Interleukins/

17. interleukin.mp.

18. exp Cytokines/

19. cytokines.mp.

20. interferon.mp.

21. exp Interferons/

22. exp Inflammasomes/

23. inflammasome.mp.

24. 16 or 17 or 18 or 19 or 20 or 21 or 22 or 23

25. 5 and 15 and 24

Embase 1974- (provider: Ovid)

Start and end date of search: 8^th^ of February 2013 (86 reports found).

Renewed search: 20^th^ of June 2013 (88 reports found).

Updated search: 3^rd^ of August 2014 (100 reports found).

1. exp phthalic acid/

2. phthalate*.mp.

3. exp plasticizer/

4. plasticizer.mp.

5. 1 or 2 or 3 or 4

6. leukocyte.mp.

7. exp leukocyte/

8. leucocyte.mp

9. exp monocyte/

10. monocyte.mp.

11. mononuclear cell/

12. exp mononuclear cell/

13. exp macrophage/

14. macrophage.mp.

15. 6 or 7 or 8 or 9 or 10 or 11 or 12 or 13 or 14

16. interleukin.mp.

17. exp cytokine/

18. cytokine.mp.

19. exp interferon/

20. interferon.mp.

21. exp inflammasome

22. inflammasome.mp.

23. 16 or 17 or 18 or 19 or 20 or 21 or 22

24. 5 and 15 and 23

Toxline (provider: US National Library of Medicine)

Start and end date of search 12^th^ to 13^th^ of February 2013 (27 reports found).

Renewed search: 20^th^ of June 2013 (27 reports found).

1. phthalate

2. plasticizers

3. #1 or #2

4. leukocyte

5. leucocyte

6. monocyte

7. mononuclear cell

8. macrophage

9. #4 or #5 or #6 or #7 or #8

10. interleukin

11. cytokine

12. interferon

13. inflammasome

14. #10 or #11 or #12 or #13

15. #3 and #9 and #14
